# Therapeutic Potential of the Natural Compound S-Adenosylmethionine as a Chemoprotective Synergistic Agent in Breast, and Head and Neck Cancer Treatment: Current Status of Research

**DOI:** 10.3390/ijms21228547

**Published:** 2020-11-13

**Authors:** Laura Mosca, Francesca Vitiello, Alessandra Coppola, Luigi Borzacchiello, Concetta Paola Ilisso, Martina Pagano, Michele Caraglia, Giovanna Cacciapuoti, Marina Porcelli

**Affiliations:** Department of Precision Medicine, University of Campania “Luigi Vanvitelli”, Via L. De Crecchio 7, 80138 Naples, Italy; laura.mosca@unicampania.it (L.M.); francesca.vitiello@unicampania.it (F.V.); alessandra.coppola@unicampania.it (A.C.); luigi.borzacchiello@unicampania.it (L.B.); concettapaola.ilisso@unicampania.it (C.P.I.); martina.pagano@unicampania.it (M.P.); michele.caraglia@unicampania.it (M.C.)

**Keywords:** S-adenosylmethionine, natural compounds, phytochemicals, head and neck cancer, breast cancer, cisplatin, doxorubicin, cancer therapy, synergistic interaction

## Abstract

The present review summarizes the most recent studies focusing on the synergistic antitumor effect of the physiological methyl donor S-adenosylmethionine (AdoMet) in association with the main drugs used against breast cancer and head and neck squamous cell carcinoma (HNSCC), two highly aggressive and metastatic malignancies. In these two tumors the chemotherapy approach is recommended as the first choice despite the numerous side effects and recurrence of metastasis, so better tolerated treatments are needed to overcome this problem. In this regard, combination therapy with natural compounds, such as AdoMet, a molecule with pleiotropic effects on multiple cellular processes, is emerging as a suitable strategy to achieve synergistic anticancer efficacy. In this context, the analysis of studies conducted in the literature highlighted AdoMet as one of the most effective and promising chemosensitizing agents to be taken into consideration for inclusion in emerging antitumor therapeutic modalities such as nanotechnologies.

## 1. Introduction

Cancer is a leading cause of death worldwide, with rapidly increasing incidence and mortality. The World Health Organization has estimated approximately 1.8 million new cases and 606,520 cancer deaths will be diagnosed in 2020 [1]. Carcinogenesis is a multistage process that leads to the dysregulation of a complex network of signaling pathways that cause uncontrolled cell growth [2]. The focus of the current treatment regimens is to work by blocking key targets within the signaling pathways that cause the transformation of a normal cell into a cancerous one. Although the use of targeted therapies based on chemotherapy drugs has brought significant improvements in the treatment of cancer, their use is limited due to the onset of adverse reactions to drug resistance, and loss of target specificity [3,4]. For instance, doxorubicin (Doxo), a widely used chemotherapy agent, frequently induces cardiomyopathy and chronic heart failure at cumulative doses [5]. Cisplatin (cDDP) is the main drug used for the treatment of head and neck squamous cancer cells (HNSCC), breast and ovarian cancer although cases of nephrotoxicity and immunosuppression by cDDP have been reported during treatment [6]. In addition, higher doses of drugs are required during chemotherapy treatment to achieve an anticancer effect, such as the initial dosage, since cancer cells develop drug resistance very early. Often, higher dosages are more likely to have serious side effects. In this light, the identification and development of powerful and better tolerated treatments, including the use of natural compounds as adjuvants in combination therapy with conventional chemotherapy drugs, are needed to address, and hopefully, solve this problem. Several physiological compounds can sensitize conventional cytotoxic therapies, decrease effective drug concentration, intensify the combined effect of both administered therapies and act as selective cytotoxic agents against cancer cells. Moreover, combined therapy, due to its multitargeted mode of action and potential synergistic behaviors, could increase the efficacy of standard chemotherapy by overcoming drug resistance and reducing toxicity and side-effects [7,8,9].

S-Adenosylmethionine (AdoMet also known as SAM or SAMe) has been extensively studied in therapy and its use, in combination with other drugs or alone, is considered as a potentially effective strategy for cancer treatment and chemoprevention [10,11,12,13,14,15,16,17,18,19,20,21,22,23,24,25,26,27,28,29]. AdoMet represents an important and naturally occurring sulfonium compound found in all mammalian cells, in which it exerts a variety of well-documented biological functions [30,31,32,33,34,35,36,37]. As a matter of a fact, AdoMet, one of the most versatile molecules present in nature, is the link to four key metabolic pathways: transmethylation, transsulfuration, polyamine synthesis and 5′-deoxyadenosyl 5′-radical-mediated biochemical transformations. The presence of the sulfonium pole makes AdoMet an extremely reactive compound, able to donate the methyl group, the adenosyl group and the aminopropyl group. Furthermore, AdoMet takes part in the biosynthesis of diphthamide, ethylene and in several post-translational modification reactions, where it donates its side chain, and is able to donate the NH2-group of the side chain during biotin synthesis. AdoMet can also exert an allosteric modulation in various enzymatic reactions. Furthermore, AdoMet in the liver acts as an important regulator of the levels of glutathione, the well-known endogenous antioxidant involved in the prevention of liver diseases [37].

The first known and well-studied metabolic reaction from the elucidation of the AdoMet structure is the transmethylation pathway where the sulfonium compound donates its methyl group to a large variety of acceptor molecules in reactions catalyzed by methyltransferases [31,34]. Epigenetic modification is the most important effect obtained from the methylation reaction and represents one of the most studied processes in cancer [38]. DNA methylation based on the methylation of CpG islands in the region of a gene promoter can inhibit the access of the transcriptional machine to chromatin resulting in silencing of gene expression. The hypomethylated pattern of cytosine methylation in CpG dinucleotides in gene regulatory regions is normally associated with carcinogenesis and plays a crucial role in cancer transformation, development and invasion. Data accumulated over the past two decades established that methylation of genes responsible for cell invasion and metastasis could be a potentially effective therapeutic target in cancer and highlighted the possibility of using the methyl donor AdoMet to suppress tumor metastases.

The antiproliferative properties of AdoMet have recently been highlighted by focusing on the identification of biological mechanisms and on the exploration of the signal transduction pathways connected to the chemopreventive activities of the sulfonium compound [10,11,12,13,14,15,16,17,18,19,20,21,22,23,24,25,26,27,28,29]. Notably, AdoMet, is an approved food supplement which can therefore be used for therapeutic purposes without the common contraindications of chemotherapy drugs. Moreover, several clinical studies to date have indicated that, at pharmacological doses, AdoMet has a low incidence of side-effects with an excellent tolerability record and with no toxic or antiproliferative effects in normal, non-tumorigenic cells [12,15,39].

A growing body of evidence indicates that combined administration of AdoMet with chemotherapy drugs improves tumoricidal effects on cancer cells. AdoMet has been found to synergize with 5-azacytidine or its deoxy-analogue (5-azaCdR) by blocking the adverse demethylating activity of these anticancer drugs while maintaining their growth suppression effects [18]. The 5-azacytidine is an epigenetic drug that inhibits DNA methylation, specifically urokinase-type plasminogen activator (uPA) and matrix metalloproteinase-2 (MMP2) promoter methylation, thus causing cancer growth inhibition and induction of cancer invasiveness, respectively. Treatment with this compound in combination with AdoMet in noninvasive and invasive breast cancer cell lines revealed that while in noninvasive MCF7 and ZR-75-1 cells, AdoMet inhibited the invasiveness induced by 5-azaCdR and potentiated its beneficial inhibitory effects on growth, in highly invasive MDA-MB-231 cells AdoMet synergized with 5-azaCdR to suppress uPA expression, thereby blocking MDA-MB-231 cell invasiveness [18]. In HeLa cells of human cervical carcinoma AdoMet in association with selenium compounds, selenomethionine, methylselenocysteine and methylseleninic acids induced inhibition of cell proliferation, migration and adhesion modulating ERK and AKT signaling pathways [24].

In MCF-7 breast cancer cells, the combined treatment of AdoMet with the autophagy inhibitor chloroquine, synergistically induces growth inhibition and apoptosis, by a caspase-dependent mechanism, and inhibits the activity of AKT and of the downstream effector mTOR. Overall, these results indicate for the first time that autophagy in these cells may represent a survival mechanism preventing AdoMet-induced apoptosis and that the sulfonium compound could be exploited in combination with chloroquine or its analogues in the treatment of breast cancer [25]. Finally, in pancreatic cancer the antimetastatic effect of gemcitabine, a structurally cytosine-like antineoplastic chemotherapy drug that inhibits DNA synthesis and ribonucleotide reductase activity was improved by the simultaneous administration of AdoMet through inhibition of the JAK2 / STAT3 pathway [28].

The aim of this article is to provide a comprehensive overview of the promising chemoprotective and synergistic anticancer effects exerted by AdoMet in combination with Doxo and cDDP on the main signaling pathways involved in cell death as well as in migration and invasion processes in breast and head and neck cancer (HNC). Comparison with promising and best studied phytochemicals currently used in synergy with Doxo in breast cancer and with cisplatin in HNC has also been discussed in this study.

## 2. Synergistic Interactions between Anticancer Drug Doxorubicin and AdoMet in Breast Cancer Cells and Comparison with Emerging Phytochemicals

Breast cancer is one of the most frequently diagnosed diseases in women. Since it is caused by multiple factors, it is currently treated in several ways. Therapies are personalized and depend on several factors, including tumor subtype, tumor stage, genomic markers, patient age, general health, menopause status, and even the presence of mutations known as BRCA1 or BRCA2 [40,41]. It is important to note that around 70% of women who develop this cancer do not have identifiable risk factors. Generally, breast cancer is classified into carcinoma in situ and invasive (infiltrating) carcinoma, and growth patterns and cytological features are used to distinguish between the two categories [42,43]. Despite the introduction of screening programs, the improvement of techniques and treatments to increase patient survival, breast cancer remains a major cause of death in women today [42,43].

One of the most effective chemotherapeutic approaches used in the treatment of breast cancer is the administration of Doxo. Doxo is an anthracycline drug commonly used in the treatment of a wide range of cancers and is a first-line chemotherapy drug used in the treatment of early and advanced breast cancer. It slows or stops the growth of cancer cells by interfering with the functions involved in DNA replication [44]. As a consequence of the growth inhibition processes, Doxo elicits a notable apoptotic response. However, the clinical usefulness of Doxo in the treatment of cancer is often limited by the development of drug resistance and various side effects including cardiotoxicity and dose-limiting myelosuppression [5]. Therefore, combination therapies of Doxo with natural molecules with antiproliferative properties capable of overcoming drug resistance and increasing therapeutic efficacy are emerging as a primary strategy [45,46,47,48,49,50,51,52].

Here, we review the latest findings in the promising research field of the potential synergistic antitumor effects of AdoMet and Doxo in the treatment of breast cancer.

Our research group has demonstrated that, in hormone-dependent CG5 and MCF-7 cell lines, the combined effect of AdoMet and Doxo was highly synergistic, while in hormone-independent MDA-MB 231 cells the effect was only additive. In CG5 cells AdoMet reduced the concentration of Doxo administrated to 1.25 µM and a similar value was found in MCF-7 and in MDA-MB 231 cells. The combination of AdoMet and Doxo induced a significant increase of the death receptor Fas and its ligand FasL, key molecules playing a crucial role in the mechanism of apoptosis [53,54,55,56]. Fas, through binding to the death domain of Fas-associated protein (FADD), mediates the recruitment and activation of the apoptosis-initiating protease, caspase-8, which in turn leads to the activation of pro-caspase 3 thus triggering the apoptotic process [21].

**Figure 1:**
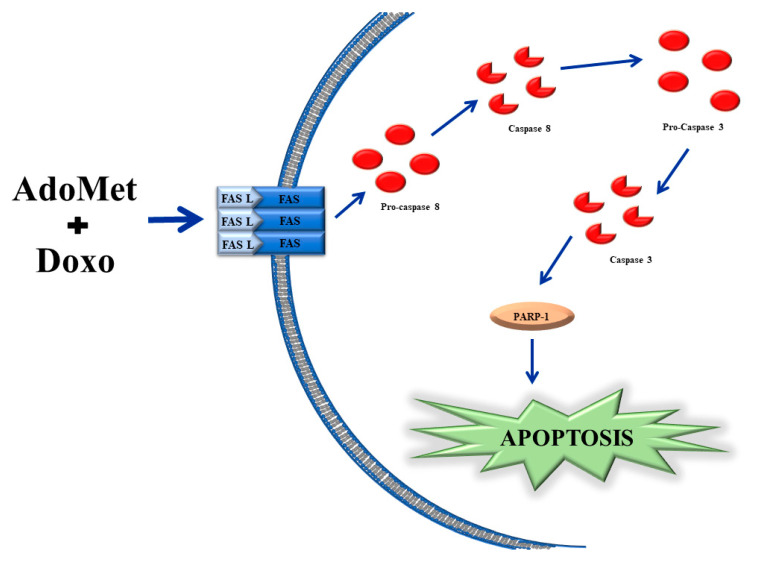
Schematic representation of the mechanism underlying the synergistic antitumor effects of S-adenosylmethionine and doxorubicin in CG5 breast cancer cells.

All together our data indicate that AdoMet synergizes with Doxo by enhancing the extrinsic apoptotic pathway. Figure 1 schematically summarizes the proposed mechanism responsible for the synergistic anticancer effect of the AdoMet/Doxo combination in CG5 breast cancer cell death.

The ability of AdoMet in enhancing the efficacy of Doxo in breast cancer cells is substantiated by evidence indicating that DNA hypomethylation plays a role in the development of Doxo resistance in human breast cancer [57].

The development of breast anticancer therapies involving natural drugs has undergone exponential growth in recent years and many natural molecules have been tested in combination with Doxo in the treatment of breast cancer. Among natural dietary products polyphenols exhibit pleiotropic mechanisms of action as they target multiple signaling pathways regulating key cellular processes thus justifying the increasing enthusiasm regarding the combination of these molecules and conventional therapy in breast cancer. The following examples illustrate the most recent literature data on the use of phenols and polyphenols to overcome doxorubicin resistance in breast cancer.

Resveratrol, a phytoalexin with pharmacological antitumor properties [58], reversed Doxo resistance in breast cancer cells by inhibiting the epithelial-mesenchymal transition and modulating SIRT1/β-catenin signaling pathway [51].

Honokiol, a naturally-occurring phenolic compound isolated from *Magnolia grandiflora* known to exert wide-ranging anticancer activity in vitro and in vivo by regulating numerous signaling pathways [59] increased the efficacy of Doxo-mediated growth suppression of MCF-7 and MDA-MB-231 cells. The findings provided mechanistic evidence that honokiol-dependent downregulation of Mucin 1, a transmembrane protein implicated in reduced survival rate and of multidrug resistance proteins responsible for acquiring Doxo resistance, increases Doxo potency [47].

Quercetin, a dietary flavonoid with antioxidant, antiproliferative and proapoptotic properties [60,61] has been found to potentiate the antitumor effect of Doxo in human breast cancer cells by increasing the intracellular drug accumulation through downregulating the expression of ATP-binding cassette transporters [49,62,63].

Curcumin, a naturally-occurring polyphenol derived from the rhizomes of turmeric plant *Curcuma longa*, with anti-inflammatory, antioxidant, antimicrobial and anticancer properties [64,65], has been reported to reverse Doxo resistance by inhibiting ATPase activity of ATP binding cassette subfamily B member 4 [52].

Recently other phytochemicals from natural sources have been utilized to improve the efficacy of Doxo in breast cancer.

Aziz and colleagues, in a paper published in 2016, reported that damnacanthal an anthraquinone extracted from the roots of *Morinda citrifolia* displayed a variety of healthy properties [66] when used in combination with Doxo enhancing the cytotoxicity of the drug in MCF-7 cells by activating apoptosis [46].

Genistein, the most common and well-known isoflavone produced in soya with potential beneficial effects on human health and possible therapeutic application in a wide range of diseases, including cancer [67] in combination with Doxo exerted a synergistic effect on MCF-7/Adr cells, a Doxo-resistant breast cancer cell line, through a mechanism involving increase in the intracellular accumulation of Doxo and downregulation of the human epidermal growth factor receptor 2/neu, a proto oncogene whose overexpression in breast cancer has been associated to chemotherapy resistance [48].

Piperlongumine (PL), a natural alkaloid from *Piper longum* L., with selective cytotoxicity against multiple cancer cells of different origins [68] synergized with Doxo to inhibit cell growth and to induce apoptosis in triple negative breast cancer cells through suppression of the JAK2-STAT3 pathway [50].

Table 1 summarizes the mechanisms of action underlying the synergistic effects of AdoMet and plant derivatives in combination with Doxo in breast cancer cells.

All these works have shown significant improvements in breast cancer treatment. Notably, the efficacy of AdoMet in sensitizing CG-5 cells toward Doxo is comparable with that of these well-investigated phytochemicals highlighting AdoMet as a potential chemopreventive and therapeutic agent to reduce breast cancer-associated morbidity and mortality.

Cancer treatment development has been greatly enhanced by the application of nanotechnology in drug delivery that has provided many potential benefits, including site-specific targeting and the ability to deliver synergistic drug combinations to the sites of drug action thereby reducing side-effects.

Recently, innovative nanocarriers have been developed to improve breast cancer therapy based on the combined treatment of natural compounds and Doxo [69,70,71,72].

The synergistic antitumor efficacy of Doxo and baicalein, a flavonoid isolated from the roots of *Scutellaria baicalensis* has been reported on Doxo resistant MCF7/Adr cells and in mice bearing MCF-7/Adr cells. Codelivery of the two drugs by nanostructured lipids decorated with hyaluronic acid showed the highest cytotoxicity and synergistic effect with respect to other different formulations resulting in the best choice to overcome the adverse reactions of Doxo and reduce its systemic toxicity [69].

The biotin-decorated poly(ethylene-glycol)-b-poly(ε-caprolactone) nanoparticles encapsulated with Doxo and quercetin, have shown significant advantages over treatment with the free drug combination, nanoparticles loaded with a single drug, or non-biotin-decorated nanoparticles in the treatment of MCF7/Adr resistant breast cancer cells, in vitro and in vivo furnishing evidence that biotin receptor-mediated tumor targeting nanoparticles encapsulating the chemotherapy drug and chemosensitizer could provide specific and efficient formulations to reverse drug resistance in human breast cancer [70].

In a study published in 2019, the effect of co-encapsulation of curcumin and Doxo in albumin nanoparticles was tested using MCF7 breast cancer cells. Concomitant administration of chemotherapy and chemosensitizer drugs led to increased intracellular accumulation of Doxo due to curcumin-induced inhibition of expression of P-glycoprotein a drug efflux pump clinically associated with the development of multidrug resistance, resulting in more efficient cell killing with respect to the sequential drug co-administration [71]. The results of this study suggest the importance of setting the modalities of concomitant administration of chemotherapy and chemo-sensitizer drugs to optimize the anticancer effects. Furthermore, very recently, it has been found that combination therapy based on curcumin-loaded biocompatible nanocarriers restored Doxo efficacy also in resistant triple negative breast cancer cells and resulted very effectively in overcoming Doxo chemoresistance mediated by P-glycoprotein [72].

Further studies are needed for the construction and encapsulation of AdoMet and Doxo in nanovectors, which will be helpful for improving the potential of the sulfonium compound as synergistic agent in combination therapy against breast cancer.

## 3. Synergistic Interactions between Anticancer Drug Cisplatin and AdoMet in Head and Neck Cancer Cells and Comparison with Emerging Phytochemicals

HNSCC are epithelial tumors that originate from several anatomic sites including skin, lip, salivary glands, sinuses, oral cavity, pharynx, and larynx. HNSCC are highly aggressive and more than 800,000 new cases are diagnosed each year. Major risk factors in developing HNC include alcohol consumption, human papilloma virus infections and tobacco use. Although surgery, radiotherapy, chemotherapy and targeted therapy are the first-line treatment options, mortality is still high. In order to improve the survival rate, significant advances are needed in the identification of new therapeutic targets [73,74,75].

cDDP is a pivotal chemotherapeutic agent, which exerts marked antitumor activity in various human solid tumors and is the standard chemotherapeutic drug to treat recurrent and metastatic HNSCC [76,77,78,79]. cDDP exerts cytotoxic effects mainly through the generation of DNA-platinum adducts responsible for DNA damage response and for induction of apoptosis in cancer cells. Toxic side effects and intrinsic or acquired drug resistance after long-term application of cDDP precluded its clinical use as a monotherapy. However, these limitations can be overcome by combination treatments of cDDP with active chemical or natural compounds able to act as synergistic and/or sensitizing agents useful to reduce the effective doses of cDDP and the related toxicity [78,79,80].

Among natural compounds, AdoMet has proven to be very effective in enhancing the cDDP anticancer effects in HNSCC cells. Our research group demonstrated that combined treatments with AdoMet and cDDP synergistically enhanced apoptosis in oral cancer cells, Cal-33 and JHU-SCC-011 by strongly decreasing the levels of uncleaved isoforms of caspase-9, caspase-6, and their target PARP with a concomitant increase of Bax/Bcl-2 ratio [27,81]. Moreover, the combination of the two drugs synergistically potentiated the activation of JNK and ERK1/2 and downregulated the expression level of Bcl-2 thus modulating two important signaling pathways involved in cDDP resistance [82,83].

In recent years, several natural compounds have been used in combination with cDDP and the synergistic activated mechanisms have been studied in HNSCC [84,85,86,87]. Noteworthy, despite the considerable benefits derived from combined therapies, none of the natural compounds reported exhibited such a strong and powerful synergistic effect as that shown by AdoMet in combination with cDDP.

Among phytochemicals, flavonoids abundant in plants, foods such as fruits and vegetables, as well as in traditional herbs, as already mentioned, are considered as the ideal candidates for cancer chemoprevention, being multitargeting and multifunctional molecules able to kill cancer cells and to reverse their multidrug resistance [84,85,86,87]. Quercetin the most abundant flavonoid found in plants with well-documented anticancer effects [60,61] synergized with cDDP in inducing apoptosis in oral squamous carcinoma cells through the Akt-IKKβ-NF-κB-xIAP axis and reduced the effective cDDP concentrations to 16.6 μM [88]. Curcumin has received great interest in the past two decades due to its biofunctional properties such as anticancer, antioxidant, and anti-inflammatory activities [64,65]. In FaDu and PE/CA PJ49 HNSCC cell lines curcumin potentiated the apoptotic process induced by cDDP by modulating ERK1/2 phosphorylation and lowered the required dose of the drug to 10 μM thus reducing its toxic adverse reactions [89]. In Hep2 human squamous laryngeal cancer cells curcumin enhanced the chemotherapeutic action of 25 μM cisplatin through activation of TRPM2 channel and mitochondrial oxidative stress and reduced cisplatin-induced drug resistance [90]. In cisplatin-resistant HNSCC cells the combined treatment of cisplatin with the curcumin analog H-4073 significantly reversed the chemoresistance and potentiated the therapeutic efficacy of cisplatin in inhibiting cell migration and increasing apoptosis [91].

Finally, two natural compounds PL and thymoquinone (TQ) were reported as very efficient molecules used in combination with cDDP in HNSCC. PL, a natural alkaloid with high pharmacological relevance as an anticancer agent synergistically increased cDDP-induced cytotoxicity in human AMC-HN3 and HN9 cells targeting the oxidative stress response and reduced to 5 μM the dose of the cisplatin required to induce apoptosis thus lowering the potential adverse effects of pharmacological chemotherapy [92]. TQ is extracted from seeds of Nigella sativa, a medicinal plant with antibacterial, antifungal, antiviral, anti-inflammatory and anticancer properties. In combination with cDDP, TQ inhibits cell viability and increase apoptosis induced by cDDP in a dose- and time-dependent manner [93]. Thymoquinone, one of the active components of the medicinal plant *Nigella sativa*, with promising potential as a therapeutic agent in the prevention and treatment of cancer, potentiated the cytotoxic effect of 5 μM cDDP in decreasing cell viability and inducing apoptosis in HNSCC [93]. Table 2 summarizes the mechanisms of action underlying the synergistic effects of AdoMet and plant derivatives in combination with cDDP in HNSCC.

Noteworthy, AdoMet reduced the concentration of cDDP needed to induce apoptosis and to inhibit migration to 0.18 μM [27,81] which is, to the best of our knowledge, the lowest concentration reported so far in combined treatments with cDPP and natural chemosensitizer compounds in HNSCC and other types of human tumor. This finding appears particularly interesting since such extremely low subtoxic concentrations of cDDP would result, in combination with AdoMet, in a much greater efficacy than its effective dosage allowing the minimization of drug toxicity without affecting its antitumor potency. It has to be pointed out, in this respect, that in non-small cell lung cancer cell lines, curcumin sensitizes cells at cDPP concentration equal to or less than 3 μM, through the downregulation of cyclin D1 expression and a substantial increase in p21 expression followed by Apaf1 and caspase-9 activation [94].

Recently, our research group demonstrated that AdoMet was not only able to sensitize HNSCC cells to cDDP-induced apoptosis, but also synergized with cDDP in reducing HNSCC cell migration [29]. In this regard, it should be noted the recently reported role of cisplatin in inhibiting the migration and invasion of nasopharyngeal carcinoma cells in vitro by repressing the Wnt/β-catenin/endothelin-1 axis via the activation of B cell translocation gene 1 [95].

Acquisition of invasive traits by tumor cells requires specific phenotypic changes associated with epithelial to mesenchymal transition (EMT), a highly regulated transdifferentiation process in which carcinoma cells lose cell-to-cell junctions and cell polarity and acquire migratory and invasive properties. EMT has been shown to play a key role in HNSCC cancer progression and metastasis [29]. We found that AdoMet was able to inhibit EMT in Cal-33 and JHU-SCC-011 cells in a dose-dependent manner by modulating TGF-β-SMAD signaling and downregulating the expression of β catenin, two potent inducers of EMT process and key mediators for metastases in other tissues during cancer progression [29]. Recently, EMT has received increasing attention for its role in cancer drug resistance and targeting EMT is now considered as a new opportunity to improve the clinical outcomes of current anticancer therapies [96,97]. In line with this evidence, the ability of AdoMet to inhibit EMT could represent an effective mechanism to reverse chemotherapeutic resistance to cisplatin further highlighting the potential therapeutic usefulness of the sulfonium compound as an adjuvant in HNSCC treatment.

**Figure 2:**
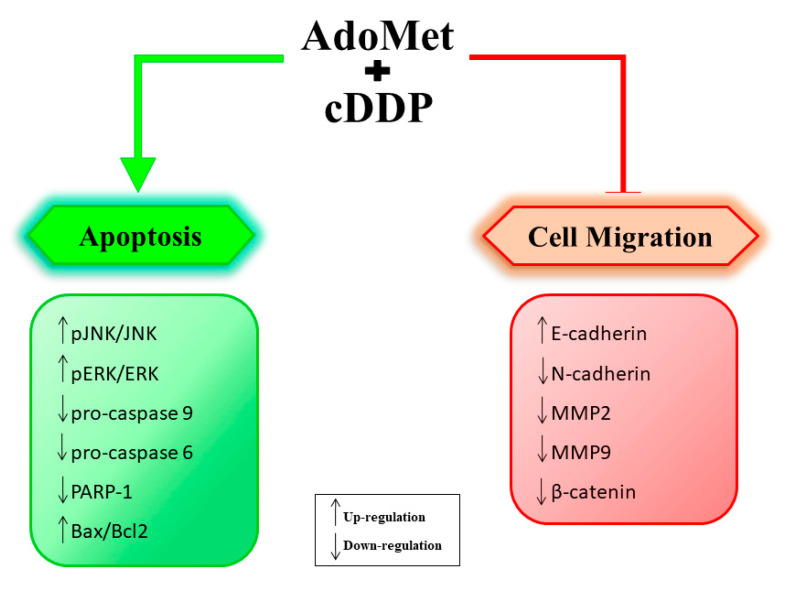
Molecular mechanisms underlying the synergistic effects of S-adenosylmethionine and cisplatin on apoptosis and cell migration in head and neck cancer cells.

Figure 2 summarizes the synergistic effects of AdoMet and cDDP in HNSCC.

## 4. Conclusions

Combination chemotherapy is the first choice for the treatment of numerous types of cancer, including breast cancer and HNSCC.

Biologic drugs are used for the treatment of numerous diseases and are the most advanced therapies available. Some natural compounds, due to their multitargeted mode of action and potential synergistic behavior could enhance the efficacy of standard chemotherapy by overcoming drug resistance and by reducing toxicity and side-effects [7,8,9]. Furthermore, the goal of biological compounds is to target diseased cells without affecting normal cells, that is not possible using conventional drugs.

AdoMet is an FDA-approved dietary supplement since 1999, and pharmaceutical preparations of this compound are available as intravenous, intramuscular, and oral forms. Maximum plasma concentrations of 0.5–1 mg/L are achieved 3–5 h after doses of AdoMet ranging from 400 to 1000 mg/day [15]. The sulfonium compound is an effective anti-inflammatory, antidepressant and analgesic molecule used in the therapeutic treatment of depression, liver disease, osteoarthritis and other pathological conditions [15,98,99]. Reviews of clinical studies to date indicate that, at pharmacological doses, AdoMet has a low incidence of side effects with a very good tolerability record. Thus, it is conceivable that AdoMet concentrations that would inhibit cancer cell proliferation, could be useful for further patient studies. Several evidences highlighted the antiproliferative potential of AdoMet and increased the scientific interest in the knowledge of biological mechanisms underlying its antitumor activity and of the targeted signal transduction pathways [10,11,12,13,14,15,16,17,18,19,20,21,22,23,24,25,26,27,28,29]. Our previous studies on breast cancer and HNSCC led us to consider AdoMet, the naturally-occurring multifunctional sulfonium compound, as a potential candidate for drug development based on its ability to modulate cancer cell growth and survival through the simultaneous regulation of multiple signaling pathways [21,22,23,24,25,26,27,28,29].

Evasion of apoptosis by malignant cells is a hallmark of cancer and induction of apoptosis by cytotoxic agents is one of the strategies adopted for the development of chemotherapy for cancer treatment. The available literature indicates that the signaling cascades for apoptotic cell death induced by many natural compounds are different among cancer cells types. Notably, AdoMet efficiently activated caspase 8 by FAS-mediated signaling in hormone-dependent breast cancer cells while it induced apoptosis in HNSCC via the intrinsic mitochondrial pathway.

The results of the present review provide a basis for further investigation of combined treatments in animal models. A greater understanding of the mechanism underlying the synergism will be useful for developing safe drug combinations and reducing the health impact of multidrug resistance.

More in-depth studies are required to improve the application of this type of therapy, such as the use of nanotechnologies that could increase the solubility and/or bioavailability of natural and synthetic compounds and bring other potential benefits into cancer therapy, including more selective and efficient targeting and further reduction of the toxicity through simultaneous administration of natural compounds and chemotherapeutic drugs. Notably, to the best of our knowledge, few studies are available in the literature regarding the formulation of SAMe-loaded nanoparticles which have been found to be an environmentally sensitive vehicle suitable for controlling drug delivery [100,101].

In conclusion the emerging results reinforce the idea that among natural compounds AdoMet could represent one of the most promising candidates as an effective adjuvant to chemotherapeutical agents to be used in combination therapy.

## Figures and Tables

**Table 1:** Effect of AdoMet and emerging phytochemicals in combination treatment with Doxo in breast cancer.

Compound	Cancer Cell Lines	Findings	Anticancer Mechanism	References
AdoMet	CG5	Synergistic activation of extrinsic apoptotic pathway	Activation of caspase 8 and caspase 3, upregulation of Fas and FasL	[21]
Resveratrol	MDA-MB-231 MCF7 MCF7/ADR	Inhibition of cell migration Inhibition of epithelial-mesenchymal transition	Modulation of Sirt1/β-catenin signaling pathway	[51]
Honokiol	MDA-MB-231 MCF7	Enhancing of Doxo-mediated growth suppression Reversion of Doxo resistance	Regulation of MUC1 and MRP1	[47]
Quercetin	MCF-7	Inhibition of cell proliferation and invasion	Downregulation of the expression of ATP-binding cassette transporters	[49,62]
Curcumin	MDA-MB-231 MCF7	Increase of Doxo efflux	Inhibition of ATPase activity of ABCB4	[52]
Damnacanthal	MCF7	Enhancing of apoptosis	Modulation of BAX/Bcl-2 pathway	[46]
Genistein	MCF7/Adr	Induction of cell cycle arrest and apoptosis. Increase of Doxo intracellular accumulation	Suppression of Her2 expression	[48]
Piperlongumine	MDA-MB-231 MDA-MB-453	Synergistic inhibition of cell growth and induction of apoptosis Synergistic suppression of xenograft tumor growth	Inhibition of JAK2-STAT3 pathway	[50]

**Table 2:** Effect of AdoMet and emerging phytochemicals in combination with cDDP in head and neck cancer.

Compound	Cancer Cell Lines	Findings	Anticancer Mechanism	References
AdoMet	Cal-33 JHU-SCC-011	Inhibition of cell proliferation Enhancing of apoptosis via intrinsic mechanism Synergistic inhibition of cell migration	Activation of caspase 6 and 9. Decrease of uncleaved PARP levels. Downregulation of Bcl-2. Increase of Bax/Bcl-2 ratio. Activation of JNK and ERK1/2 signaling.	[27,29,81]
Quercetin	Tca-8113 SCC-15	Enhancing of cDDP-induced apoptosis	Activation of caspase cascade. Inhibition of Akt-IKKβ-NF-κB-xIAP axis	[88]
Curcumin	FaDu PE/CA PJ49	Enhancing of cDDP-induced apoptosis Enhancing of cDDP-induced cell death Enhancing of antitumor and antiangiogenic effects of cDDP	Modulation of ERK1/2 expression Activation of TRPM2 channel and mitochondrial oxidative stress Inhibition of JAK/STAT3, FAK, Akt, and VEGF signaling pathways	[89,90,91]
Piperlongumine	AMC-HN3 HN9 cells	Enhancing of cDDP-mediated apoptosis	Puma and PARP activation	[92]
Thymoquinone	UMSCC-14C	Enhancing of apoptosis and inhibition of cell viability	Downregulation of Bcl-2 and increase of p53, caspase 6 and 9 expression	[93]

## Abbreviations

AdoMet	S-adenosylmethionine
HNSCC	head and neck squamous cell carcinoma
HNC	head and neck cancer
5-azaCdR	5-deoxy-azacytidine
uPa	urokinase-type plasminogen activator
MMP2	matrix metalloproteinase-2
Doxo	doxorubicin
PL	piperlongumine
cDDP	cisplatin
TQ	thymoquinone
EMT	epithelial to mesenchymal transition
